# Repetitive Transcranial Magnetic Stimulation (rTMS) as a Promising Treatment for Craving in Stimulant Drugs and Behavioral Addiction: A Meta-Analysis

**DOI:** 10.3390/jcm11030624

**Published:** 2022-01-26

**Authors:** Aurélia Gay, Julien Cabe, Ingrid De Chazeron, Céline Lambert, Maxime Defour, Vikesh Bhoowabul, Thomas Charpeaud, Aurore Tremey, Pierre-Michel Llorca, Bruno Pereira, Georges Brousse

**Affiliations:** 1 University Department of Psychiatry and Addiction, CHU St-Etienne, CEDEX 2, 42055 Saint-Étienne, France; defourmaxime@gmail.com (M.D.); vikesh.bh@gmail.com (V.B.); 2 TAPE Laboratory, EA7423, Jean Monnet University, 42100 Saint-Étienne, France; 3 Clermont Auvergne INP, CHU Clermont-Ferrand, CNRS, Institut Pascal, Université Clermont Auvergne, 63000 Clermont-Ferrand, France; jcabe@chu-clermontferrand.fr (J.C.); idechazeron@chu-clermontferrand.fr (I.D.C.); pmllorca@chu-clermontferrand.fr (P.-M.L.); gbrousse@chu-clermontferrand.fr (G.B.); 4 Biostatistics Unit (DRCI), CHU Clermont-Ferrand, 63000 Clermont-Ferrand, France; clambert@chu-clermontferrand.fr (C.L.); bpereira@chu-clermontferrand.fr (B.P.); 5 Service d’Addictologie et Pathologies Duelles, CHU Clermont-Ferrand, 63000 Clermont-Ferrand, France; tcharpeaud@chu-clermontferrand.fr (T.C.); atremey@chu-clermontferrand.fr (A.T.)

**Keywords:** addiction, craving, dorsolateral prefrontal cortex, eating disorder, gambling disorder, transcranial magnetic stimulation, substance use disorder

## Abstract

Addiction is a mental disorder with limited available treatment options. The therapeutic potential of repetitive transcranial magnetic stimulation (rTMS) on it, by targeting craving in particular, has been explored with heterogenous results. This meta-analysis uses updated evidence to assess overall rTMS efficacy on craving, differential effects between addiction types clustered into three groups (depressant (alcohol, cannabis, opiate), stimulant (nicotine, cocaine, methamphetamine), and behavioral addiction (gambling, eating disorder)), and stimulation settings. Studies on substance use, gambling, and eating disorders are included, with unrestricted stimulation settings, by searching the PubMed, Embase, PsycINFO, and Cochrane databases up to 30 April 2020. A total of 34 eligible studies (42 units of analysis) were identified. Because of highly significant heterogeneity in primary results, a sensitivity analysis was performed on a remaining sample of 26 studies (30 units of analysis). Analyses performed using random effects model revealed a small effect size favoring active rTMS over shamTMS stimulation in the reduction in craving. We found a significant difference between addiction types, with a persistent small effect only for stimulant and behavioral groups. In these groups we found no difference between the different combinations of target and frequency of stimulation, but a significant correlation between number of sessions and craving reduction. In conclusion, efficacy of rTMS on craving in stimulant and behavioral addiction was highlighted, but recommendations on optimal stimulation settings and its clinical application await further research.

## 1. Introduction

Drug addiction can be defined as a chronically relapsing disorder, characterized by compulsion to seek and take a drug, loss of control in limiting its intake, and emergence of a negative emotional state (e.g., dysphoria, anxiety, irritability) when access to the drug is prevented [1]. In 2013, DSM-5 [2] defined substance use disorders (SUDs) over a range from mild to moderate to severe, with the severity of an addiction depending on how many of the established 11 specific diagnosis criteria apply. The latest version of these diagnostic criteria also includes “non-substance-related disorders”, defined as addictive disorders not involving ingestion of a psychoactive substance. Currently, gambling disorder in DSM-5 [2] and gaming disorder in the 11th Revision of the International Classification of Diseases (ICD-11) [3] are the only conditions included in international classifications. In an extended definition of behavioral addiction (BA), eating disorders (EDs) (anorexia nervosa, bulimia nervosa, and binge eating disorder) have also been deemed forms of addiction [4,5].

Addiction is a chronic disorder that is very costly to affected patients and to society in general [6]. According to the World Health Organization (WHO), the use of tobacco, alcohol, and illicit drugs contributes significantly to the global burden of disease, is implicated in over 12% of mortality worldwide, and is the leading cause of preventable death [7]. Behavioral addictions are also pervasive and disabling. Gambling disorder has adverse impacts such as disrupted family and marital relationships, financial difficulties, mental and physical health problems, and diminished life fulfillment [8]. Bulimia nervosa is associated with an increased mortality, especially by suicide [9], and EDs affect the quality of life of both patients and their families, and individuals with these disorders have particularly high rates of health service use [10,11].

To date, available treatment options for addictive behaviors remain limited, and long-term success rates are poor [12]. Meta-analytical studies suggest that across substances, combined pharmacological treatment and behavioral intervention may increase clinical success and ameliorate clinical attendance and patient retention [13,14,15]. For pharmacotherapy, many different substances have been tested [16]. However, the results of these trials have fallen short of expectations [17,18,19,20], not all addictive disorders have empirically validated treatments, and available pharmacotherapies do not meet overall clinical requirements. There is therefore a need to explore novel approaches to treating substance-related and addictive disorders. Craving is one treatment target that can be usefully addressed.

Craving is a core clinical symptom of addiction. It has received considerable research attention for several decades [21]. Craving is defined as a pressing, urgent, and irrepressible desire to give way to an addictive behavior, motivated by internal and external cues, resulting in loss of control in most cases [22]. It also includes the expectation of previously experienced effects of a psychoactive drug, whether these are its positive effects or the relief of its negative effects, or both [23]. This key symptom has been extensively studied in drug addiction and eating disorders [24,25] and in other behavioral addiction such as gambling disorder [26]. There are currently various experimental measurements of this state: standardized questionnaires, visual analogue scale (VAS), and non-verbal physiological measures [27,28]. Drug craving is considered an important risk factor for relapse in patients with addictive disorder, as shown for example in alcohol [29,30], cocaine [31,32], or gambling [33], and alleviating craving has been considered a beneficial target to curb addictive behavior [28].

Neuroimaging studies in humans have shown that craving is underpinned by activation of the reward and motivation circuits. These studies show that the main structures involved are the nucleus accumbens, dorsal striatum, amygdala, hippocampus, insula, and the prefrontal cortex (PFC), including dorsolateral prefrontal cortex (DLPFC), anterior cingulate cortex (ACC), and medial orbitofrontal cortex (OFC) [34,35,36,37,38].

Neurobiologically, addiction is associated with disturbances in the brain’s reward, stress, and executive function systems [1,39,40]. Notably, repetition of intake and binges are associated with the replication of a dopamine-firing pattern through the mesocorticolimbic pathways, leading to the development of incentive salience and drug-seeking habits [41] and to neuroadaptations resulting in decreased dopamine secretion [42]. The craving and deficits in executive function, in the so-called preoccupation/anticipation stage, could involve the dysregulation of key afferent projections from the PFC and insula to the basal ganglia and extended amygdala. This neurobiological model of addiction provides an approach to medication development. The therapeutic potential of brain stimulation techniques has been suggested [1].

Repetitive transcranial magnetic stimulation (rTMS) is a noninvasive brain stimulation technique that leads to cerebral neuromodulation through a modification of cortical excitability, of blood flow to the area [43], of the frequency of neuronal discharge [44], and of the release of neurotransmitters such as dopamine [45,46]. In addition to its cortical action, TMS is claimed to act remotely on deeper structures, via brain circuits and interhemispheric connections [47,48,49]. An alternative to the conventional figure-of-eight rTMS coils is the so-called H-coil system [50], developed to reach deeper brain regions [51] and whose derived therapeutic application has been called “deep transcranial magnetic stimulation” (dTMS) [52]. High-frequency (HF) stimulation (≥5 Hz) is considered to have excitatory effects on the targeted cortical excitability, whereas low-frequency (LF) stimulation (≤1 Hz) is reported to have inhibitory effects [53]. These effects can outlast the stimulation period. Following these “classic” protocols, new TMS paradigms have been developed such as “theta burst stimulation” (TBS), delivered continuously (cTBS) (inhibitory effects) or intermittently (iTBS) (excitatory effects) [54]. Numerous studies have shown that rTMS produces significant clinical effects in patients with various neurological and psychiatric disorders [55]. One line of research that has emerged since the early 2000s examines the efficacy of rTMS on addiction and related disorders by targeting craving in particular. Transcranial direct current stimulation (tDCS) is another non-invasive brain stimulation method that generates a low intensity electric field between two electrodes, increasing neuronal excitability under the anodal but decreasing it under the cathodal electrode [56]. tDCS is also currently being investigated as a potential treatment for SUDs with encouraging results, but we chose to focus on rTMS in our work [57].

As regards targets, particular attention has been paid to the DLPFC in neuromodulation studies in addiction. First, its stimulation could increase dopamine excretion in mesolimbic and mesostriatal pathways, through its interconnection with the ventral tegmental area (VTA) and ventral striatum [46,58,59], and thus could redress drug-induced dopaminergic dysfunction [60]. Second, DLPFC repetitive stimulation could correct diminished functioning of the prefrontal cortex including DLPFC, ventromedial prefrontal cortex (VMPFC), and ACC described in imaging studies in addictive disorders [61], and presumably underlining diminished cognitive and behavioral control, and a higher tendency to cue-induced relapse [62,63]. More recently, other approaches have been proposed, such as inhibition of the medial PFC to decrease striatal and insula activity, thereby weakening drug-related craving and attenuating frontostriatal reactivity to substance-related cues [64].

Eight meta-analyses [24,57,65,66,67,68,69,70] have focused on neuromodulation and addictive disorders but with various inclusion criteria and some differences in conclusions, notably concerning differences in efficacy according to addiction type. On the one hand, previous studies that included several addiction types found no difference as regards indication, but some found differences for stimulation settings. Jansen et al. (2013) was the first to show that stimulation (including also tDCS studies; 17 studies overall) on the DLPFC can decrease craving in patients with SUD and food addiction, without significant differences between various substances of abuse or between substances of abuse and food [24]. These results were replicated by Song et al. (2018), who included 44 studies focusing on DLPFC activation [57]. Two other meta-analyses, limited to SUD, pointed to differences only for stimulation settings. Enokibara et al. (2016) found active rTMS stimulation to outperform placebo only for right DLPFC stimulation [65], contrary to Zhang et al. (2019), who found that only excitatory rTMS of the left DLPFC significantly reduced craving [70]. On the other hand, four other meta-analyses, which restricted their scope to certain types of addiction, failed to replicate positive results for all of them: Maiti et al. (2016) focused on HF stimulation in alcohol and nicotine use disorder [68], Lowe et al. (2017) on food craving [66], including rTMS and tDCS studies, Ma et al. (2019) on psychostimulants (cocaine, amphetamine, and methamphetamine) [67], and Mostafavi et al. (2020) on alcohol [69]. These studies supported rTMS efficacy on craving in nicotine use disorder [68], psychostimulants [67], and food [66], but not in alcohol use disorder [68,69].

Studies so far thus tend to support rTMS as a novel, safe anti-craving therapeutic intervention, but results are still controversial, in particular concerning preferred indication, with heterogenous results according to addiction type. Additionally, behavioral addictions were not systematically included and were restricted to eating disorder/food addiction. Moreover, the studies are characterized by a wide variation in relation to the rTMS intervention protocols (target and frequency of stimulation, number of pulses per session, number of sessions), and the optimal treatment settings remain to be specified [49,71]. The primary aim of our meta-analysis was thus to evaluate the updated evidence regarding the effects of rTMS compared with sham stimulation (shamTMS) on craving in substance-related and addictive disorders, including all behavioral addiction, and to better define indication according to addiction type. Second, we aimed to better define optimal stimulation settings as regards target (side and location) combined with frequency of stimulation (high versus low) and method for locating it (use of neuronavigation or not), number of sessions, and number of pulses (per session and total).

## 2. Materials and Methods

This meta-analysis was conducted and reported in conformance with the Preferred Reporting Items for Systematic Reviews and Meta-Analysis (PRISMA) statement [72]. The Cochrane handbook was used as a methodological reference [73]. We registered the protocol in the International Prospective Register of Ongoing Systematic Reviews (systematic review registration—PROSPERO 2020: CRD42020114671).

### 2.1. Search Strategy

We identified articles for inclusion in this review by searching the PubMed, Embase, PsycINFO, and Cochrane databases up to 30 April 2020, limiting our search to articles published in English and German (one article). Our enquiry was constructed following the PICO method (Population, Intervention, Comparison, Outcome). The key items we used in our search were as follows: “P”, substance-related and addictive disorders/substance use disorder/addiction to drugs, alcohol and nicotine/dependence/substance abuse/eating disorder/gambling disorder/behavioral addiction; “I”, rTMS/transcranial magnetic stimulation/deep TMS); “C”, shamTMS/placebo/control group; and “O”, craving.

### 2.2. Inclusion Criteria for the Selection of Studies

To control for the placebo effect, only randomized controlled trials (RCTs) and controlled clinical trials (CCTs) were included in our meta-analysis. Craving reduction had to be an outcome measure, either primary or secondary.

Concerning type of participants, we included studies examining adult human subjects of either sex with a diagnosis of substance-related and addictive disorders, including behavioral addictions, meeting DSM-5 or former DSM-IV-TR substance abuse or dependence or ICD-10 criteria. By equating eating disorders with behavioral addiction, we included in our meta-analysis studies examining subjects with anorexia nervosa (AN), bulimia nervosa (BN), and binge eating disorder (BED), but not healthy subjects experiencing strong food craving. As comorbidity is very common in addictive disorders, we did not exclude studies of subjects with psychiatric comorbidity.

Studies had to evaluate the effect of rTMS (figure-of-eight or double-cone coil) or deep TMS (dTMS), irrespective of stimulation settings (including theta burst stimulation) and method used to localize the target. rTMS/dTMS could be performed alone or as an add-on to usual pharmacological treatment or psychotherapy. The outcome measure was change in craving after last rTMS session from baseline (pre rTMS), measured by different craving assessment tools.

The studies included were not restricted by date of publication, craving assessment tool, number of stimulation sessions, or site of stimulation. Studies evaluating tDCS effect were excluded from our meta-analysis.

### 2.3. Study Selection and Data Collection

The relevant studies were selected in a stepwise manner. A manual search and screening of the bibliography of the selected studies were performed in addition to the computerized screening. Duplicate searches were eliminated. First, all studies were screened based on title and abstract. Second, the full texts of all the studies from this selection were read, and studies were included in the meta-analyses if all inclusion criteria, determined before the literature search was performed, were met. Three authors independently performed study selection (A.G., G.B., and J.C.) with disagreements resolved by discussion in consultation with the statistics advisor (B.P.), referring to guidelines published by Cochrane Collaboration [73]. Extracted data for meta-analysis comprised study design, participants, substance or behavior involved, site of stimulation, method for localization, frequency of stimulation, number of stimulation sessions, total pulse per session, and standardized effect sizes for the effect of stimulation on craving levels. Graphically reported data were extracted from the figures by one author (A.G.) and one statistics advisor (C.L. or B.P.). In cases of disagreement, a third extraction was made by I.D.C. or J.C. For descriptive purposes, Supplementary Data were collected: motor threshold, assessment time-points, and outcome measures other than craving.

“Study” was considered as unit of design instead of the “unit for analyses”. Studies in which two tools were used to assess craving, or in which two or more stimulation settings were compared, were considered separately as two “units of analysis”. If studies compared the effect of rTMS on craving using exposure to neutral and addiction-related stimuli, we selected cue-induced craving only, as this is considered as the most ecological measure of craving [71,74]. When reported data were insufficient for data analysis, authors were contacted to retrieve the data.

### 2.4. Data Analysis

Baseline characteristics are summarized for each study sample and reported as mean and standard deviation (SD) or median and inter-quartile range, and number (%) for continuous and categorical variables, respectively. The meta-analysis took account of between- and within-study variability. To address the non-independence of data due to study effect, random effects models [75] were preferred to the usual statistical tests to assess the standardized paired mean differences (SMDs) of craving and their 95% confidence intervals. Hedge’s *g* SMDs were estimated to assess the difference between baseline (T0) and last follow-up (T1) evaluation in craving levels, between active and shamTMS stimulation. The analyses were performed (i) on post-session craving change when this was reported in studies or (ii) on the calculation of craving change from baseline (T1–T0) with standard deviation of the paired mean difference between T0 and T1 estimated using the formula: [SD^2^_T0_ + SD^2^_T1_ − (2 × 0.5 × SD_T0_ × SD_T1_)]. Means and standard deviations were compiled when available or were estimated when median and interquartile range were reported [76]. When standard deviation was not available, an estimation according to available standard deviations (for other studies) was calculated. The cross-over nature of the within-subject study was ignored, and the sample was treated as two separate groups. This method provides a more conservative estimate of the effect size and allows comparison of between- and within-subject designs. Hedge’s *g* is considered to be a conservative estimate, which is useful for studies with small sample sizes, and the results may be interpreted as reflecting a small (*g* = 0.2–0.5), medium (*g* = 0.5–0.8), or large effect (*g* > 0.8) [77].

Heterogeneity in the study results was assessed by forest plots and the *I*^2^ statistic, the most common metric for measuring the magnitude of between-study heterogeneity, and one which is easily interpretable. *I*^2^ values range between 0 and 100% and are typically considered low for <25%, moderate for 25–50%, and high for >50% [78]. Publication bias was assessed by funnel plots, confidence intervals, and with the Egger regression test as a formal statistical test.

To check the robustness of the results, sensitivity analyses were performed, excluding studies that were not evenly distributed around the base of the funnel plots. Furthermore, as dTMS can be considered as a different technique of rTMS, a sensitivity analysis without the studies using these techniques was also conducted.

Meta-regression and subgroup analyses were then performed to compare the influence of the different addiction types on effect size for studies retained after sensitivity analyses. Because of the various addiction types included, we opted to categorize them into three subgroups, based on their expected effect for SUD: depressant (alcohol, cannabis, and opiates) or stimulant (nicotine, cocaine, methamphetamine), and individualizing the behavioral group (eating and gambling disorder). As study design (crossover versus parallel design) may mediate stimulation effects [79], meta-regression was performed to identify the potential influence of independent variables on SMD. For an addiction type for which a significant effect was highlighted, the influence of target (location and side) combined with stimulation setting (HF/LF), method used for target localization (neuronavigation/other methods), and number of sessions (single versus multiple) were assessed. The relationships between number of sessions, number of pulses per session, total number of pulses, and craving change from baseline only in the rTMS group, were also evaluated (Table A1 in Appendix A).

Statistical analyses used Stata software (version 15, StataCorp, College Station, TX, USA). Two-sided type I error was fixed at 5%.

## 3. Results

### 3.1. Search Results

**Figure 1:**
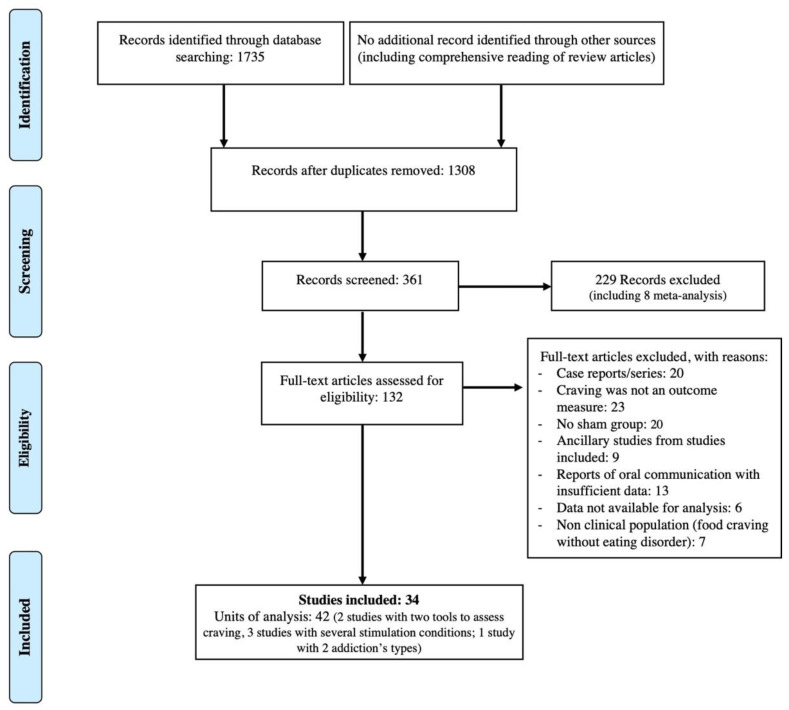
PRISMA flow diagram for the study selection process.

After screening on PICO criteria, the initial literature search identified 132 potentially eligible studies for assessment and inclusion in the meta-analysis. In addition to these studies, review articles were thoroughly read to ensure no study was missed. The PRISMA flow diagram depicts the full selection process (Figure 1). For diverse reasons, 98 studies were excluded at this stage—namely, case reports or series; studies not mentioning craving as an outcome measure; non-controlled trials (no shamTMS stimulation group, of which three comparing different stimulation sites, one comparing new population with a subsample of another study, and three with standard drug treatment as a control group, without a shamTMS coil); ancillary studies from studies included in the meta-analysis, presenting imaging, biological, or cognitive data; studies for which only conference abstracts were available and with too many missing data or too much uncertainty concerning study overlap and with no author information; and trials in subjects with high food craving without ED. In eight studies, detailed statistical data were not sufficient to calculate a standardized mean difference. The corresponding authors were contacted by e-mail and asked to provide the data necessary for the meta-analysis; only two authors responded, despite reminders. Two authors could not be contacted because their e-mail addresses were incorrect or not provided in the article. Thus, 34 studies were finally included in the meta-analysis (42 units of analysis) (see in Appendix A Table A1 for descriptive data of included studies and their main results, including other outcome measures than craving).

In 11 studies, data were extracted totally or partly from figures [80,81,82,83,84,85,86,87,88,89,90].

Of the 34 studies included in the meta-analysis, 1 concerned both alcohol and cocaine, 10 alcohol, 8 nicotine, 1 cannabis, 1 cocaine, 6 methamphetamine, 1 opiate, 3 eating disorder (2 BN or “eating disorder not otherwise specified”—bulimic type (EDNOS-BN), 1 AN), and 3 gambling disorder (Table A1 in Appendix A).

dTMS was used in three studies in the meta-analysis [86,91,92].

**Figure 2:**
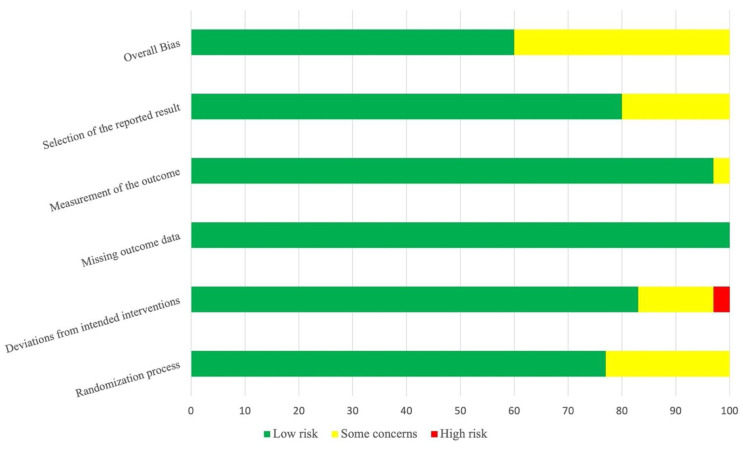
Risk summary of bias. For each risk of bias item, proportion of studies included in the meta-analysis (*k* = 42) with low, high, or unclear risk according to authors’ judgments.

The quality assessment of the studies included in the meta-analysis is presented through a risk of bias graph in Figure 2, showing the proportion of studies with each judgment (“low risk”, “some concerns”, “high risk” of bias) for each risk of bias item. Detailed risk of bias for each study is presented in Supplementary Materials (Figure S1).

### 3.2. Main Results

#### 3.2.1. Overall Effect of rTMS and Sensitivity Analysis

**Figure 3:**
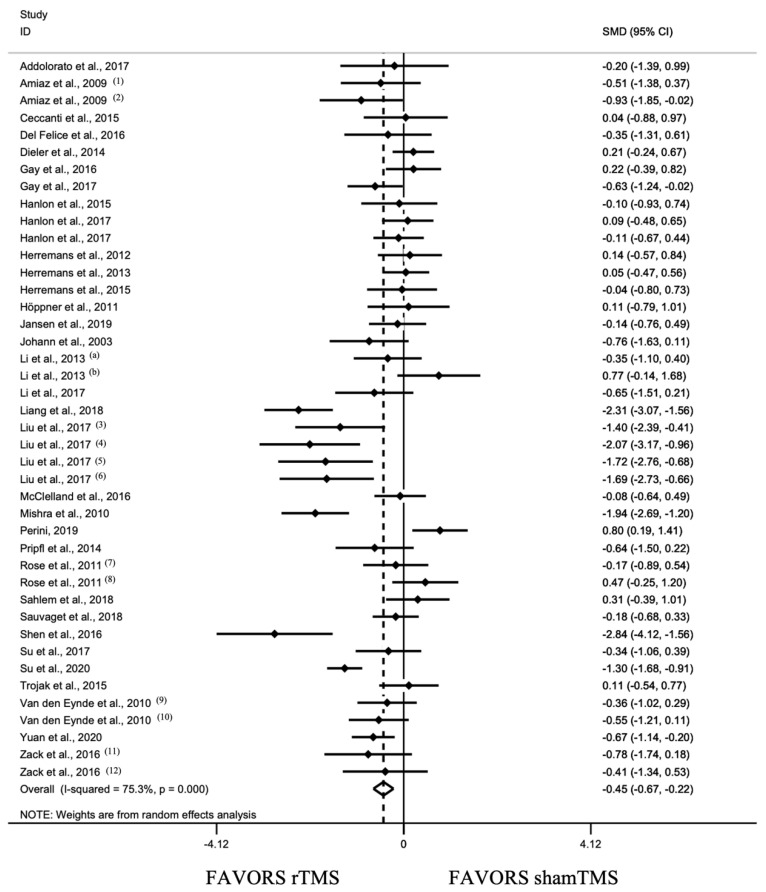
Forest plot for all included studies pooled together using random effects model (34 studies, 42 units of analysis) [80,81,82,83,84,85,86,87,88,89,90,91,92,93,94,95,96,97,98,99,100,101,102,103,104,105,106,107,108,109,110,111,112,113]. Heterogeneity: Q = 165.67, df = 41, *p* < 0.001, *I*^2^ = 75.3%. (1), craving evaluated by Visual Analog Scale (VAS); (2), craving evaluated by sTCQ; (3), high frequency left DLPFC stimulation; (4), high frequency right DLPFC stimulation; (5), low frequency left DLPFC stimulation; (6), low frequency right DLPFC stimulation; (7), low frequency stimulation; (8), high frequency stimulation; (9), craving evaluated by VAS; (10), craving evaluated by FCQ-s; (11), high frequency MPFC stimulation; (12), low frequency right DLPFC stimulation; (a), [103]; (b), [89].

This analysis revealed a pooled standardized effect size (Hedge’s *g*) of −0.445 (95% CI: −0.667, −0.224), indicating an overall small effect size favoring active stimulation over shamTMS stimulation (*z* = 3.94, *p* < 0.001) (Figure 3). The test for heterogeneity was significant (*I*^2^ = 75.3%). The Egger regression test was not significant for overall studies initially included (*k* = 42, *t* = −1.38, *p* = 0.175).

**Figure 4:**
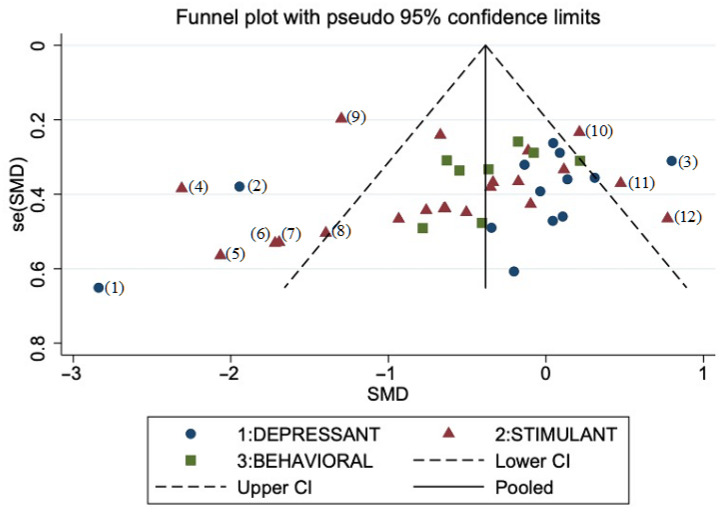
Funnel plot for publication bias of the included studies (42 units of analysis). (CI, confidence interval; SE, standard error; SMD, standard mean difference). Type of addiction: depressant (alcohol, cannabis, opiate); stimulant (nicotine, cocaine, methamphetamine); behavioral (eating disorder, gambling disorder). Outlier studies excluded from sensitivity analysis: (1), [108]; (2), [106]; (3), [86]; (4), [84]; (5–8), [85]; (9), [110]; (10), [95]; (11), high frequency arm from [88]; (12), [89].

Based on visual inspection of a funnel plot (Figure 4), we excluded 12 units of analysis and performed sensitivity analyses on the remaining units of analysis (*k* = 30). The test for heterogeneity was no longer significant (*I*^2^ = 0%). This analysis revealed a pooled standardized effect size of *g* = −0.228 (95% CI: −0.355, −0.102), indicating an overall small effect size favoring active stimulation over shamTMS stimulation (*z* = 3.53, *p* < 0.001).

After excluding outlier studies, the funnel plot was almost symmetrical, indicating minimal publication bias across the studies. Sensitivity analysis after excluding dTMS studies showed no impact on overall rTMS effect. Further analyses were performed on our restricted sample after sensitivity analysis (*k* = 30).

#### 3.2.2. Analyses between Addiction Type Groups

**Figure 5:**
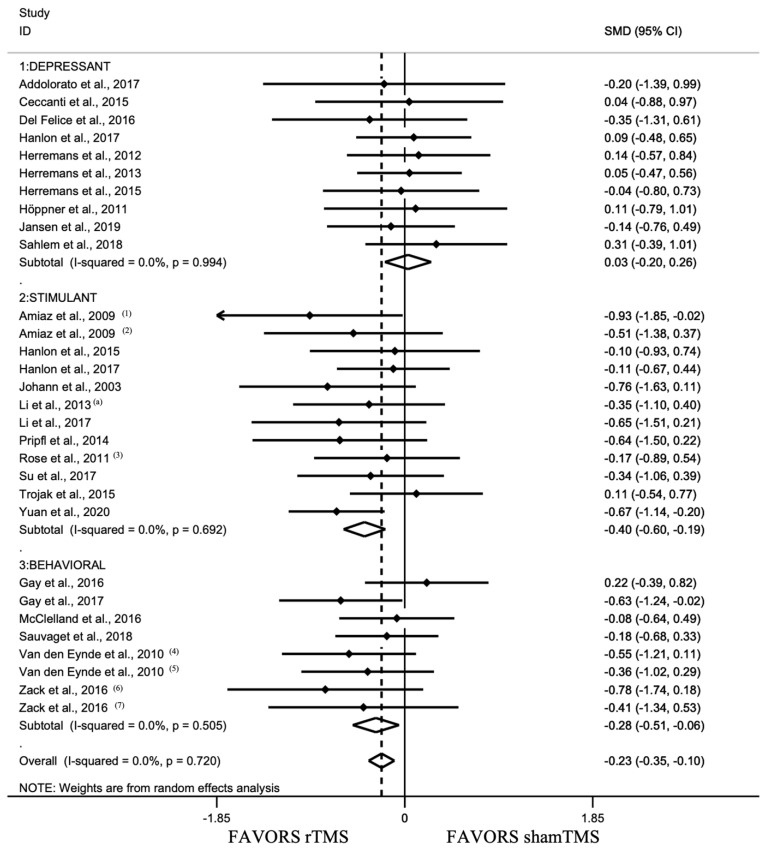
Forest plot for the subgroup analysis by addiction type’s group (depressant: alcohol, cannabis, opiate [81,82,91,92,94,98,99,100,101,107]; stimulant: nicotine, cocaine, methamphetamine [80,87,88,90,93,98,102,103,104,109,111]; and behavioral: eating disorder, gambling disorder [89,96,97,105,112,113]) using random effects model (without excluded studies, 30 units of analysis). Heterogeneity: Q = 24.18, df = 29, *p* = 0.72, *I*^2^ = 0%. Test for overall effect: Hedge’s *g* = −0.228 (95% CI: −0.355, −0.102), *z* = 3.53, *p* < 0.001. (1), craving evaluated by Visual Analog Scale (VAS); (2), craving evaluated by sTCQ; (3), low frequency stimulation; (4), craving evaluated by VAS; (5), craving evaluated by FCQ-s; (6), high frequency MPFC stimulation; (7), low frequency right DLPFC stimulation; (a), [103].

As stated above, given the various addiction types included (eight different types), we opted to cluster them into three groups: depressant, stimulant, and behavioral (Figure 5 and Table A1 in Appendix A).

Meta-regression analyses showed a significant difference on SMD between addiction groups (F(2, 27) = 3.92, *p* = 0.032) with a significant difference only between depressant and stimulant groups (*t* = −2.74, *p* = 0.011). A pooled standardized effect size of *g* = 0.032 (95% CI: −0.195, 0.259) was estimated for the depressant group, indicating no effect of active versus shamTMS stimulation (*z* = 0.28, *p* = 0.781). A pooled standardized effect size of *g* = −0.396 (95% CI: −0.601, −0.191) was observed for the stimulant group and of −0.284 (95% CI: −0.511, −0.056) for behavioral addiction, indicating for both a small effect size favoring active over shamTMS stimulation (respectively, *z* = 3.78, *p* < 0.001 and *z* = 2.45, *p* = 0.014) (Figure 5).

**Figure 6:**
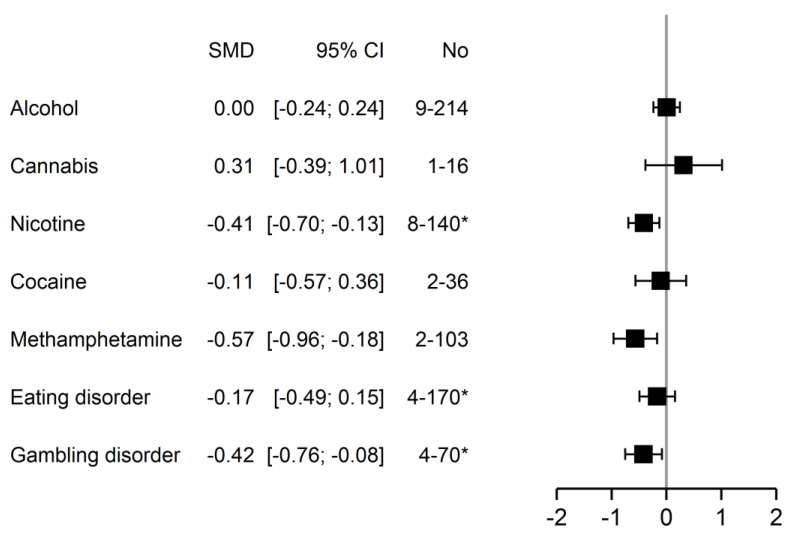
Subgroup analysis by addiction type using random effects model (without excluded studies, 30 units of analysis). (CI, confidence interval; No, number of units of analysis—number of patients included; SMD, standard mean difference). * Indicating difference between number of units of analysis and number of studies, with the following data: nicotine, 7–119; eating disorder, 3–133; gambling disorder, 3–61.

For descriptive purposes, results by addiction type are presented in Figure 6. Subgroup analyses showed a significant effect only for nicotine, methamphetamine, and gambling disorder, indicating for all a small effect size favoring active over shamTMS stimulation, except for methamphetamine, with a medium effect size (nicotine: *z* = 2.88, *p* = 0.004; gambling: *z* = 2.44, *p* = 0.015; methamphetamine: *z* = 2.83, *p* = 0.005).

### 3.3. Analysis of Stimulation Settings

Analyses of stimulation settings are presented only for stimulant and behavioral groups, pooled together (*k* = 20), as no significant effect was observed in the depressant group.

We found no significant difference between crossover and parallel studies (*t* = 0.34, *p* = 0.736).

As regards stimulation settings, meta-regression analyses showed no significant difference on SMD between method of target localization (use of neuronavigation or not) (*t* = 0.14, *p* = 0.894) and number of sessions (single versus multiple) (*t* = 0.16, *p* = 0.875). Meta-regression between different combinations of target and frequency could not be carried out because numbers of studies per group were too small. Subgroup analyses are presented for their descriptive interest in Table 1.

In these two groups pooled together (stimulant and behavioral addictions), only in the rTMS group, we found no correlation between craving change from baseline and number of pulses per session (r = −0.037, *p* = 0.881) but found a marginal association with the total number of pulses (r = −0.454, *p* = 0.059) and a significant correlation between craving change from baseline and number of session (r = −0.45, *p* = 0.046).

### 3.4. Safety

Of all the studies, 33 reported side effect occurrences. rTMS was well-tolerated and the most common side effects were benign discomfort and headache, less frequently insomnia (two studies) and dizziness.

## 4. Discussion

This updated random effects meta-analysis is the first to include all addiction types (SUD and behavioral addiction, including gambling disorder). After excluding outlier studies, our results reveal a small though significant effect size (Hedge’s *g* = −0.228) favoring real rTMS over shamTMS stimulation in the reduction in craving and for the first time a significant difference between addiction types, with a persistent significant effect only for stimulant and behavioral addictions. In these groups we found a significant correlation between effect size of rTMS intervention and the number of sessions and a trend for the total number of pulses.

The overall effect observed is in line with results from previous meta-analyses [24,57,68], not replicated by other studies [65,70]. Direct comparisons are limited by methodological differences, as our study is the only one to include all addiction types, with no restriction on stimulation settings. In particular, Enokibara et al. (2016) and Zhang et al. (2019) restricted their scope to substance use disorder, whereas we show a significant effect in the behavioral addiction group [65,70]. Our size effect was consistent with the meta-analysis of Song et al. [57], but it was smaller than in two previous studies [24,68] that found a medium effect size (respectively, Hedge’s *g* = 0.476 and 0.75). These earlier meta-analyses included a smaller number of studies, 9 rTMS studies for Jansen et al., 10 for Maiti et al., and again several methodological differences. Jansen et al. (2013) evaluated effects of both rTMS and transcranial direct current stimulation (tDCS) [24], and Maiti et al. (2016) narrowed their scope to SUD (alcohol and nicotine) and high frequency stimulation [68].

Our meta-analysis is the first to show a significant difference between addiction type, with a persistent significant effect only for stimulant and behavioral addiction. Given the various addiction types included (eight different types), we opted to cluster them into three groups, based for substance use disorder on their classical observed effects: depressant versus stimulant and separating behavioral addiction. These results are consistent with subgroup analyses in previous studies. Thus, our lack of rTMS efficacy on depressants, mainly alcohol use disorder (AUD) (9 units of analysis out of 10), but significant effect on the stimulant group (nicotine, cocaine, and methamphetamine) corroborate the subgroup analyses of Maiti et al. (2016), with no effect on AUD but a persistent effect for nicotine [68]. They are also in line with results from meta-analyses focusing on one addiction type [67,69]. Both evaluated rTMS and tDCS effects, respectively, on alcohol craving, with no significant effect found [69], and psychostimulants (cocaine, amphetamine, methamphetamine) with a large size effect only for the rTMS group. Song at al. (2019) and Zhang et al. (2019) found a persistent significant effect in alcohol subgroup analysis but with a smaller effect than for stimulant drugs (nicotine, cocaine, and methamphetamine) [57,70]. Concerning behavioral addiction, our study is the first to include gambling disorder. Although limited to three studies, our results emphasize for the first time the potential of rTMS to reduce craving in this addiction, in which no pharmacological treatment is yet validated. Some previous studies addressed food craving and also found a positive effect of rTMS [24,57,66], but this concept only partially covers eating disorders and limits comparison with our results. Food craving is associated with some forms of overeating behaviors, binge-related eating disorders or obesity [114], but also involves healthy subjects who experience frequent food craving [115,116], who lie outside the scope of this meta-analysis on addictive disorders. We note that our positive results for rTMS on craving in behavioral addictions are strengthened by the methodological quality of the studies included and a low heterogeneity, with no need to exclude outliers. A total of 5 studies out of 6 were assigned low overall risk of bias, versus 7 out of 13 for depressant and 9 out of 16 for stimulant groups.

Differences between addiction types in our meta-analysis could be partly explained by heterogeneity in stimulation settings, such as target and number of sessions. Nevertheless, some authors point out that outside shared psychobiological substrates, there are substantial differences in the neurobiological and behavioral mechanisms underlying different addictions, across drug classes [117] and between substances, particularly AUD and behavioral addictions [118]. Notably, psychostimulants cause more pronounced deficits in impulse control in humans and are associated with complete loss of control in animal models [117]. Most studies still targeted DLPFC, leading most directly to an increased control, which could partly explain rTMS efficacy in this group.

Given the high heterogeneity in stimulation settings, we set out to identify an optimal protocol for the target frequency combination, method of localization, number of sessions, and number of pulses (per session and total). We opted to perform these analyses only in stimulant and behavioral addiction groups but failed to identify optimal combinations of target and stimulation frequency because of insufficient numbers of studies for several variables. Nevertheless, for these groups (stimulant and behavioral addictions), in subgroup analysis, only left DLPFC stimulation showed a persistent favorable effect, mainly with activation variables (10 studies versus 1 for inhibition variables), unlike right DLPFC or other targets, keeping in mind the smaller number of studies in other groups. These findings are in line with the results of Zhang et al. (2019) in SUD [70] and Ma et al. (2019) in psychostimulants [67]. They conflict with those of Enokibara et al. (2016) in SUD, who showed a significant effect only after right DLPFC stimulation [65], and with the conclusions of Jansen et al. in favor of right stimulation despite no statistical difference between left and right stimulation in SUD and eating disorders [24]. Discrepancies between included studies could explain this difference in results. In our meta-analysis, DLPFC was mainly chosen, but attention has been paid to novel targets such as the medial prefrontal cortex (MPFC), which could be a more direct pathway to the limbic system [80]. They showed no favorable effect in our results, but the number of studies was small and comprised several variables. A recent controlled study with rTMS targeting medial prefrontal and anterior cingulate cortex in alcohol use disorder showed positive results on craving and alcohol consumption [119].

To go further, craving is a multidimensional phenomenon, which can be modelled on the interplay between three subcomponents—namely, cognitive craving related to cognitive abilities (related to frontal regions); automatic craving, linked to cue response and implicit processes (related to limbic structures); and physiological craving, corresponding to bodily perceptions and related to withdrawal symptoms (related to insular pathways) [120]. According to the target chosen, stimulation could act differently on these different subcomponents and optimally in individualized protocols. Thus, most studies have focused on cognitive craving and control, but targets such as MPFC or insula would respectively act more effectively on automatic or physiological craving.

Our meta-analysis found for stimulant and behavioral groups no additional benefit of neuronavigation to localize targets, contrary to data in depression [121]. However, this result was limited by the fact that most studies still did not use this method in our sample (only 6 used neuronavigation over 20 studies). It would have been interesting to consider the intensity of stimulation in our analysis of stimulation settings, but we were restricted by the limited number of studies included. The comparison between theta burst protocols versus classical ones was also precluded by the fact that only three studies used the first one after sensitivity analysis.

Unlike Song et al. (2019), we found no superiority of multiple versus single sessions, but these studies varied in number (from 4 to 20) and frequency of sessions, with a moderate statistical heterogeneity [57]. In line with the results of Song (2019) and Zhang (2019), we found a correlation between effect size and number of sessions and a trend for the total number of pulses [57,70]. A greater number of sessions and total number of pulses were associated with a greater craving reduction after rTMS intervention. We still lack detailed knowledge on neurobiological mechanisms of rTMS effects in addictive disorders. Nevertheless, translation of findings into animal models or other neurological or psychiatric disorders [122] and results from open-label studies in addiction also suggest that increasing the number of stimulation sessions might help to strengthen the effects of rTMS on the underlying mechanism regulating addictive behaviors, thereby improving its effectiveness [118].

Assessment of clinical rTMS application in addictive disorders with long-lasting effects would optimally require long-term evaluation, combined with usual treatment. When compared or associated with usual treatment, rTMS had an additive positive effect on CBT [95] or nicotine replacement therapy [111] in nicotine use disorder. Only eight studies in our meta-analysis evaluated long-lasting effects after repeated rTMS sessions, with positive results for half of them, at 1 month [106] and 6 months [92] in alcohol use disorder, at 3 months in nicotine [95], and at 3 weeks in methamphetamine use disorder [90]. However, long-lasting neurobiological effects of rTMS on reward and executive networks remain unknown. The cognitive and motivational changes that develop with addiction are associated with long-term changes in brain functions [1] and there is still no evidence that even repeated rTMS sessions could durably modulate these changes. Even so, we can also hypothesize that the “downregulated craving and improved control” therapeutic interval induced by rTMS may offer the possibility of breaking the addiction cycle by enabling patients to gain more benefit from standard drug treatment and rehabilitation programs. Further protocols could also evaluate the efficacy of maintaining rTMS sessions after successful response to initial stimulations, as proposed in depression [123] or in a recent multicenter randomized control trial in tobacco use disorder [124]. Long-term positive results of these protocols have also been suggested by an observational study in a large cohort in cocaine use disorder [125].

Our work has some limitations. Studies featured high statistical and methodological heterogeneity, but the consistency of our findings was improved by sensitivity analyses, and secondary analyses were performed to assess which variables could influence results. Nevertheless, the small number of studies included for the evaluation of some variables rule out any definite conclusions. As regards methodological aspects, numerous studies were single-blind, with poor shamTMS quality (e.g., coil angulation) in some studies, and craving outcome in rTMS studies in addictive disorder has been associated with the quality of shamTMS stimulation [115]. Allocation concealment or conditions of self-reported craving assessment were not specified in most studies. Self-report of craving was mainly used for outcome assessment, with various tools (VAS, standardized questionnaires), and is subject to socially desirable responding. Finally, patients’ characteristics (e.g., age, sex, motivation) are important to consider when assessing the effects of neuromodulation on craving in SUDs [74] and could not be controlled in the absence of individual data.

To conclude, this meta-analysis was performed on a broader sample of studies than in previous works on this topic, including for the first time all types of addiction, and confirms previous positive results of rTMS effects on craving in addictive disorders, with a small though significant effect favoring treatment. We show a significant difference between addiction groups, with a persistent effect on stimulant (nicotine, cocaine, methamphetamine) and behavioral addiction (eating disorder and gambling disorder). Practical recommendations are still difficult to make because of a high heterogeneity among study protocols and lack of long-term evaluation. Further studies are needed in a larger sample to establish the most effective treatment settings, using repeated sessions, combined or compared with usual treatments, and evaluating the rTMS effect on craving but also on other markers of addiction severity. Long-term effects should be assessed and maintenance protocols for responders considered. Homogenization in methods is needed, but in view of clinical heterogeneity of the addict population, a single neuromodulation protocol may well not fit all subjects. Further work should be conducted with a strategy more closely tailored to subjects’ profiles, particularly as regards the target, including also neurophysiological measures.

## Figures and Tables

**Table 1:** Subgroup analysis on stimulation settings (target and frequency, method of localization, number of sessions (single versus multiple)) for stimulant (nicotine, cocaine, methamphetamine) and behavioral (eating disorder, gambling disorder) groups (*k* = 20).

			Effect Size			Heterogeneity	
	*k* *	Hedge’s *g* (Random Effect)	CI 95%	*Z*-Statistic	*p*	*p*	*I* ^2^
**Target and frequency**							
HF Left DLPFC	12	−0.396	−0.603; −0.190	3.76	<0.001	0.656	0
LF Left DLPFC	1	−0.670	−1.142; −0.198	2.78	<0.001	-	-
LF Right DLPFC	3	−0.120	−0.488; 0.249	0.64	0.524	0.637	0
HF MPFC	1	−0.781	−1.744; 0.182	1.59	0.112	-	-
LF MPFC	2	−0.108	−0.570; 0.355	0.46	0.648	0.976	0
LF SFG	1	−0.175	−0.892; 0.542	0.48	0.633	-	-
**Method of localization**							
No neuronavigation	14	−0.352	−0.531; −0.173	3.86	<0.001	0.678	0
Neuronavigation	6	−0.328	−0.620; −0.037	2.21	0.027	0.436	0
**Number of sessions**							
Single	14	−0.354	−0.540; −0.169	3.74	<0.001	0.934	0
Multiple	6	−0.321	−0.687; 0.045	1.72	0.086	0.121	42.6

* Units of analysis: DLPFC, dorsolateral prefrontal cortex; MPFC, medial prefrontal cortex; SFG, superior frontal gyrus; HF, high frequency; LF, low frequency.

## Supplementary Materials

The following are available online at https://www.mdpi.com/article/10.3390/jcm11030624/s1, Figure S1: Detailed risk of bias for each study.

## Appendix A

**Table A1:** Characteristics of studies included in the meta-analysis (*k* = 34).

Studies	Design	Population	Stimulation Settings				Method of Craving Assessment and Other Outcome Measures ^2^ ^*^ Craving as Primary Outcome	Main Results
			No. of Sessions ^1^ Total Pulses/Session	Frequency (Hz) Intensity (% of RMT)	Stimulation Site Method for Locating Target	Coil Type of shamTMS		
**Depressant group**								
**Alcohol**								
Mishra et al., 2010 [106]	RCT, SB	45 M, 30 real/15 shamTMS, detoxified	10 1000	10 Hz 110%	R DLPFC NS	Figure-of-8 ShamTMS coil	ACQ-NOW *, before, after, and 1 month after last session	Significant post-rTMS reduction
Höppner et al., 2011 [81]	RCT, SB	19 F, 10 real/9 shamTMS, detoxified	10 1000	20 Hz 90%	L DLPFC 10–20 syst	Figure-of-8 45° angulation and shifting	OCDS * Mood (HDRS, BDI) Attentional blink	No significant effect on craving and mood
Herremans et al., 2012 [99]	RCT, SB,	31 inpatients (36 included), 15 real/16 shamTMS, 21 M/10 F, detoxified	1 1560	20 Hz 110%	R DLPFC Neuronavigation	Figure-of-8 90° angulation	OCDS * Before, after, and the 3 days following rTMS in natural setting	No significant effect on craving (immediate or delayed)
Herremans et al., 2013 [100]	RCT, SB, crossover, 1 week washout	29 inpatients (50 included), 19 M/10 F, detoxified	1 1560	20 Hz 110%	R DLPFC Neuronavigation	Figure-of-8 90° angulation	OCDS Go/NoGo test	No significant effect on craving Effect on IIRTV on Go/NoGo
Herremans et al., 2015 [101]	Phase 1: RCT, DB, 1 session Phase 2: NC	26 (Phase 2: 23), 13 real/13 shamTMS, 17 M/9 F, detoxified	Phase 1: 1 Phase 2: 15, for 4 days 1560	20 Hz 110%	R DLPFC Neuronavigation	Figure-of-8 90° angulation	Craving cue-induced: TLS *, before rTMS, day1 and 7 General craving: AUQ and OCDS, before rTMS, day 7 fMRI, before rTMS, day1 and 7	No significant effect on cue-induced craving Significant reduction in general craving after phase 2
Ceccanti et al., 2015 [92] dTMS	RCT, DB	18 M, 9 real/9 shamTMS, detoxified for 10 days	10 1500	20 Hz 120%	MPFC 5 cm	H coil ShamTMS coil	VAS (cue-induced) Alcohol consumption, cortisolemia, and prolactinemia Before, after rTMS and each month to 6 months	Significant reduction in craving (maintained at 1 month), alcohol consumption, cortisolemia, and prolactinemia
Del Felice et al., 2016 [94]	RCT, SB	17 inpatients (20 included), 8 real/9 shamTMS, 13 M/4 F During detoxification	4 (2/week, 2 weeks) 1000	10 Hz 100%	L DLPFC 10–20 syst	Figure-of-8 ShamTMS: wooden panel under coil	VAS Alcohol intake, EEG, Stroop, and Go/NoGo tasks Before and after rTMS sessions, at 1 month	No effect on craving and alcohol intake Significant reduction in fast EEG frequencies Effect on Stroop and Go/NoGo
Addolorato et al., 2017 [91] dTMS	RCT, DB	11, 5 real/6 shamTMS (12 M/2 F included)	12 (3/week, 4 weeks) 1000	10 Hz 100%	Bilat DLPFC 5.5 cm	H coil ShamTMS: blank session	OCDS DAT by SPECT TLFB, STAI, Zung self-rating depression scale	No effect on craving Decrease in DAT availability and alcohol intake, effect on STAI state
Hanlon et al., 2017 ** [98]	RCT, SB, crossover, 7–14 days washout	24 non-treatment-seeking alcohol-dependent, 17 M/7 F	1 3600	cTBS 110%	L FP 10–20 syst	Figure-of-8 ShamTMS coil	VAS (cue-induced) fMRI before and after cTBS (change in MPFC–striatal connectivity)	No effect on craving Decreased evoked BOLD signal in left OFC, insula, and lateral sensorimotor cortex
Jansen et al., 2019 [82]	CT, SB	39, 19 real/20 shamTMS, 26 M/13 F, Subjects sober for at least 3 weeks 36 HC	1 3000	10 Hz 110%	R DLPFC Neuronavigation guided by fMRI	Figure-of-8 90° angulation	AUQ before and after the emotional reappraisal task, after rTMS Emotion regulation and related brain activity using fMRI	No effect on craving In AUD patients: reduced self-reported emotions to positive and negative images, reduced right DLPFC activity but no significant effect of rTMS on reappraisal-related brain function
Perini et al., 2019 [86] dTMS	RCT, DB	56 (45 finished sessions), 29 real/27 shamTMS (23/22 finished), treatment-seeking alcohol-dependent patients	15 (5/week, 3 weeks) 1500	10 Hz 120%	Insula bilat and overlaying areas excluding ant PFC	H coil, H8 ShamTMS coil	AUQ cue-induced before each session; PACS during rTMS and follow-up (week 1, 2, 4, 8, 12) Consumption during rTMS, at the end of sessions and during follow-up; CGI and CRPS-SA during rTMS follow-up Structural and rsMRI before and after rTMS sessions	Decrease in craving and drinking measures but with no difference between real and shamTMS rTMS Difference in rs insula connectivity after treatment between real and shamTMS groups
Cannabis								
Sahlem et al., 2018 [107]	RCT, DB, crossover, 1 week washout	16 (2 subjects withdrew before first session), 13 M/3 F	1 4000	10 Hz 110%	L DLPFC Beam F3 method	Figure-of-8 ShamTMS coil	MCQ (cue-induced) prior, during, after, and 15 min after the completion of rTMS	No effect on craving Feasibility and safety validated
Opiate								
Shen et al., 2016 [108]	RCT	20 M, 10 real/10 shamTMS, long-term addicts	5 2000	10 Hz 100%	L DLPFC NS, no MRI	Figure-of-8 90° angulation	VAS * (cue-induced) Before, after 1st and last session	Significant effect on craving at day 1 and 5
**Stimulant group**								
**Nicotine**								
Johann et al., 2003 [102]	RCT, DB, crossover, 2 consecutive days	11, 2 M/9 F, motivated to quit	1 1000	20 Hz 90%	L DLPFC NS	Figure-of-8 45–90° angulation	VAS *	Significant craving reduction
Amiaz et al., 2009 [93]	RCT, DB, 4 arms: real/shamTMS, smoke/neutral cue	48, 26 real/22 shamTMS (smoke cue: 12/9), 21 M/27 F, ≥20 cig/day	10 and maintenance phase: 6 over 1 month 1000	10 Hz 100%	L DLPFC 5 cm	Figure-of-8 ShamTMS coil	Cue-induced craving * by VAS and sTCQ, Consumption and nicotine dependence (FTND) Before, after rTMS and at 6 months	Significant effect on cue-induced-smoke craving, on consumption and dependence No difference at 6 months
Rose et al., 2011 [88]	RCT, crossover, 3 visits (1 Hz, 10 Hz, and shamTMS)	15, 8 M/7 F, ≥20 cig/day, with good cue reactivity	1 at each frequency 1 Hz: 450/10 Hz: 4500	1 and 10 Hz 90%	SFG 10–20 syst	Figure-of-8 ShamTMS: motor cortex stimulation	Shiffman–Jarvik questionnaire (cue-induced, neutral/smoke) * Cigarette evaluation questionnaire	Cue-induced craving increase at 10 Hz but decrease if neutral cue
Li et al., 2013 [103]	RCT, DB, crossover, 1 week washout	14 (16 included), 10 M/4 F, non-treatment-seeking	1 3000	10 Hz 100%	L DLPFC 6 cm	Figure-of-8 ShamTMS coil	QSU-B (cue-induced, neutral/smoke) *	Significant effect on craving, correlated with dependence severity
Pripfl et al., 2014 [87]	RCT, cross over, 1 week washout	11 (14 included), 5 M/6 F, abstinent for 6 h	1 1200	10 Hz 90%	L DLPFC Neuronavigation	Figure-of-8 ShamTMS: vertex stimulation	5 points LS (cue-induced) * EEG	Significant reduction in craving and EEG delta power
Diehler et al., 2014 [95]	RCT, DB Add-on a 6-sessions group CBT, rTMS at meetings 3 to 6	74, 38 real/36 shamTMS, 40 M/34 F	4 (2/week) 600	iTBS 80%	R DLPFC 10–20 syst	Figure-of-8 ShamTMS: 45° angulation and 60% RMT	QSU * before CBT and after last rTMS session Abstinence rate at 3, 6, and 12 months	No effect on craving Significant effect on abstinence rate at 3 months
Trojak et al., 2015 [111]	RCT, DB Phase 1: rTMS + NRT (2 weeks) Phase 2: NRT only (4 weeks) Phase 3: follow-up (6 weeks)	37, 18 real/19 shamTMS, 20 M/17 F, motivated to quit, at least 2 unsuccessful attempts to quit	10 360	1 Hz 120%	R DLPFC Neuronavigation	Figure-of-8 ShamTMS coil	VAS, FTCQ-12, and QSU Abstinence rate Before and after rTMS, week 6 and 12	No significant effect of add-on rTMS on craving but effect on abstinence rate, without lasting effect
Li et al., 2017 [104]	RCT, SB, crossover, 1 week washout	11, 5 M/6 F	1 3000	10 Hz 100%	L DLPFC 6 cm	Figure-of-8 ShamTMS coil	VAS (cue-induced) Resting state fMRI	No effect on craving Decreased fALFF in the right insula and thalamus and temporal connectivity between L DLPFC and L OMPFC
Cocaine								
Hanlon et al., 2015 [80]	RCT, SB, crossover, 7–14 days washout	11, 9 M/2 F, non-treatment-seeking	1 3600	cTBS 110%	L MPFC 10–20 syst	Figure-of-8 ShamTMS coil	VAS * fMRI	Significant decrease in craving and striatum and ant insula activity
Hanlon et al., 2017 ** [98]	RCT, SB, crossover, 7–14 days washout	25, 12 M/13 F, non-treatment-seeking chronic cocaine users abstinent for 48 h	1 3600	cTBS 110%	L FP 10–20 syst	Figure-of-8 ShamTMS coil	VAS (cue-induced) fMRI before and after cTBS (change in MPFC–striatal connectivity)	No effect on craving Decreased evoked BOLD signal in the caudate, accumbens, anterior cingulate, orbitofrontal and parietal cortex
**Methamphetamine**								
Li et al., 2013 [89]	RCT, SB, crossover, 1 h washout	18, 10 MA dependent and 8 healthy controls, 4 M/14 F	1 900	1 Hz 100%	L DLPFC 6 cm	Figure-of-8 45° angulation	VAS (cue-induced, neutral/MA) * during rTMS session	For MA dependent only: significant craving increase
Liu et al., 2017 [85]	5 arms (10 Hz P3 = shamTMS, 10 Hz L DLPFC, 10 Hz R DLPFC, 1 Hz L DLPFC, 1 Hz R DLPFC)	50 M, detoxified for the last 2 months	5 10 Hz: 2000 1 Hz: 600	1 Hz/10 Hz 100%RMT	L DLPFC R DLPFC 10–20 syst	Round coil ShamTMS condition = P3	VAS (cue-induced) * prior to rTMS stimulation, 30 min after rTMS on day 1 and on day 5	Significant decrease in craving after either at left or right side, both high and low frequency rTMS, but not after shamTMS condition
Su et al., 2017 [109]	RCT, DB	30 M, 15 real/15 shamTMS	5 1200	10 Hz 80%	L DLPFC 5 cm	Figure-of-8 90° angulation	VAS (cue-induced) * Cognitive functions HDRS, HARS, PSQI	Significant decrease in craving, improvement in verbal learning, memory and social cognition
Liang et al., 2018 [84]	RCT, DB	48 M, 24 real/24 shamTMS	10 (12 days) 2000	10 Hz NS	L DLPFC NS, no MRI	NS	VAS (cue-induced) Withdrawal symptoms, quality of sleep, depression, and anxiety	Significant craving reduction Improvement in sleep, depression, and anxiety Withdrawal symptoms reduction in both groups
Su et al., 2020 [110]	RCT, DB	126, 70 real/56 shamTMS, 106 M/20 F	20 (4 weeks) 900	iTBS 100%	L DLPFC 10–20 syst	Figure-of-8 180° angulation	VAS (cue-induced) * at baseline and after each 5 sessions Sleep quality at baseline and after sessions, cognitive functions: at baseline, 1 month, and 12 months	Significant craving reduction Improvement in sleep quality and cognitive functions
Yuan et al., 2020 [90]	RCT, DB	73 M, 37 real/36 shamTMS	10 600	1 Hz 100%	L DLPFC 5 cm	Figure-of-8 90° angulation	Craving (cue-induced) Impulse inhibition (2-choice odd-ball task) After 1 session, 24 h after 10 sessions, and at 3 weeks follow-up	Significant craving decrease and improvement in response inhibition, both lasting 3 weeks after treatment
**Behavioral group**								
**Eating Disorder**								
Van den Eynde et al., 2010 [112]	RCT, DB	38, 17 real/20 shamTMS, 5 M/33 F BN or EDNOS-BN, fasting 2 h before	1 1000	10 Hz 110%	L DLPFC 5 cm	Figure-of-8 ShamTMS coil	VAS (cue-induced, FCT) * and FCQ-S No. of binges over the 24 h after rTMS	Significant diminution of cue-induced craving and No. of binges but no effect on FCQ-S
Gay et al., 2016 [96]	RCT, DB	47 F, 23 real/24 shamTMS BN	10 1000	10 Hz 110%	L DLPFC 6 cm	Figure-of-8 ShamTMS coil	VAS (cue-induced, FCT) before and after first and last rTMS session No. of bingeing and purging episodes in the 15 days following last session, MADRS	No significant effect on cue-induced craving or binge episode
McClelland, 2016 [105]	RCT	49 F, 21 real/28 shamTMS 28 AN-R, 21 AN-BP Mean BMI: 16.5	1 session 1000	10 Hz 110%	L DLPFC Neuronavigation	Figure-of-8	After FCT, combination of AN-related experiences evaluated by VAS of which urge to restrict * Mood, temporal discounting (TD), and salivary cortisol Before, after rTMS, and 24 h following	No significant effect of rTMS but a trend on core AN symptoms, maintained at 24 h, and on TD No effect on cortisol
**Gambling disorder**								
Zack et al., 2016 [113]	RCT, DB, 3 * 3 (rTMS, cTBS, shamTMS), crossover, 1 week washout	9 M, non-treatment-seeking	1 of each rTMS: 450 cTBS: 900	10 Hz et cTBS 80%	rTMS: MPFC cTBS: R DLPFC Neuronavigation	rTMS: double cone coil cTBS: figure-of-8 ShamTMS: vertex stimulation	VAS (cue-induced, slot machine) Cognitive tests: DDT, Stroop test Amount of money and frequency of gambling	Significant effect of rTMS only on craving No effect on impulsive choice and decrease in control with rTMS and cTBS No effect on gambling behavior
Gay et al., 2017 [97]	RCT, DB, crossover, 1 week washout	22, 14 M/8 F, treatment-seeking	1 3008	10 Hz 110%	L DLPFC Neuronavigation	Figure-of-8 ShamTMS coil	VAS (cue-induced) * Gambling behavior before and 7 days after rTMS	Significant effect on craving, no effect on gambling behavior
Sauvaget et al., 2018 [89]	RCT, DB, crossover, 1–2 weeks washout	30	1 360	1 Hz 120%	R DLPFC Beam F3 method	Figure-of-8 ShamTMS coil	VAS (cue-induced) * GACS-desire factor, heart rate, blood pressure	No effect on craving and other outcomes

10–20 syst, International 10–20 System; ACQ-NOW, Alcohol Craving Questionnaire; AN-BP, anorexia nervosa binge/purge type; AN-R, anorexia nervosa restrictive type; AUD, alcohol use disorder; AUQ, Alcohol Urge Questionnaire; BDI, Beck Depression Inventory; Bilat, bilateral; BMI, body mass index; BN, bulimia nervosa; CBT, cognitive behavioral therapy; CCT, controlled clinical trial; CGI, Clinical Global Impressions scale; CO, carbon monoxide; CRPS-SA, Comprehensive Psychopathological Rating Scale; Self-Rate; cTBS, continuous theta burst stimulation; DAT, dopamine transporter; DB, double blind; DDT, delay discounting task; DLPFC, dorsolateral prefrontal cortex; dTMS, deep transcranial magnetic stimulation; EDNOS-BN, eating disorder not otherwise specified—bulimic type; EEG, electroencephalogram; F, female; fALFF, amplitude of low frequency fluctuation; FCQ-S, Food Craving Questionnaire-State; FCT, food challenge task; fMRI, functional magnetic resonance imaging; FTCQ-12, French version of the 12-item Short Form of the Tobacco Craving Questionnaire; FTND, Fagerström Test for Nicotine Dependence; GACS, Gambling Craving Scale; HARS, Hamilton Anxiety Rating Scale; HC, healthy control; HDRS, Hamilton Depression Rating Scale; IIRTV, intraindividual reaction time variability; iTBS, intermittent theta burst stimulation; L, left; LS, Likert scale; M, male; MADRS, Montgomery–Asberg Depression Rating Scale; MPFC, medial prefrontal cortex; NRT, nicotine replacement therapy; NS, non-specified; NSc, numerical scale; OCDS, obsessive-compulsive drinking scale; OFC, orbitofrontal cortex; OMPFC, orbital middle prefrontal cortex; PACS, Penn Alcohol Craving Scale; PFC, prefrontal cortex; PG-YBOCS, Yale Brown Obsessive Compulsive Scale adapted for Pathological Gambling; PSQI, Pittsburgh Sleep Quality Index; QSU-B, Questionnaire of Smoking Urges-Brief; R, right; RCT, randomized controlled trial; RMT, resting motor threshold; rsMRI, resting state magnetic resonance imaging; SB, single blind; SFG, superior frontal gyrus; SPECT, single photon emission computed tomography; STAI, State-Trait Anxiety Inventory Scale; sTCQ, short version of the Tobacco Craving Questionnaire; TLFB, timeline followback interview; TLS, Ten-point Likert scale; U, urinary; VAS, visual analog scale. cTBS, 3 burst at 50 Hz applied at 5 Hz; iTBS, 3 burst at 50 Hz, 2 s every 10 s. ^1^ session frequency detailed only when different from usual daily protocol. ^2^ time of measure detailed only when different from pre/post rTMS. ** same study.
