# Supplementary material for: Repetitive Transcranial Magnetic Stimulation (rTMS) as a Promising Treatment for Craving in Stimulant Drugs and Behavioral Addiction: A Meta-Analysis
Source: J Clin Med. 2022 Jan 26;11(3):624. doi: 10.3390/jcm11030624 (PMC8836499; doi:10.3390/jcm11030624)
Supplement: Supplementary file 1 [file jcm-11-00624-s001.zip › jcm-1544935-supplementary.pdf]

Study	Randomization process	Deviations from intended interventions	Missing outcome data	Measurement of the outcome	Selection of the reported result	Overall
Mishra et al., 2010	?	?	+	+	+	!
Höppner et al., 2011	+	+	+	+	?	!
Herremans et al., 2012	+	+	+	+	+	+
Herremans et al., 2013	+	+	+	+	+	+
Herremans et al., 2015	+	+	+	+	+	+
Ceccanti et al., 2015	+	+	+	+	?	!
Del Felice et al., 2016	+	+	+	+	+	+
Addolorato et al., 2017	+	+	+	+	+	+
Hanlon et al., 2017	?	+	+	+	?	!
Jansen et al., 2019	?	+	+	+	+	!
Perini, 2019	+	+	+	+	+	+
Sahlem et al., 2018	+	+	+	+	+	+
Shen et al., 2016	+	?	+	+	?	!
Johann et al., 2003	?	?	+	+	+	!
Amiaz et al., 2009	+	+	+	+	+	+
Rose et al., 2011	?	?	+	?	+	!
Li et al., 2013a	+	+	+	+	+	+
Dieler et al., 2014	+	+	+	+	+	+
Pripfl et al., 2014	?	+	+	+	+	!
Trojak et al., 2015	+	+	+	+	+	+
Li et al., 2017	+	+	+	+	+	+
Hanlon et al., 2015	?	+	+	+	?	!
Hanlon et al., 2017	?	+	+	+	?	!
Li et al., 2013b	+	–	+	+	+	!
Liu et al., 2017	+	+	+	+	+	+
Su et al., 2017	+	+	+	+	?	+
Liang et al., 2018	+	+	+	+	+	!
Su et al., 2020	+	+	+	+	+	+
Yuan et al., 2020	+	+	+	+	+	+
Van den Eynde et al., 2010	+	+	+	+	+	+
Gay et al., 2016	+	+	+	+	+	+
McClelland et al., 2016	+	+	+	+	+	+
Zack et al., 2016	+	?	+	+	+	!
Gay et al., 2017	+	+	+	+	+	+
Sauvaget et al., 2018	+	+	+	+	+	+

Figure S1. Detailled risk of bias for each study, for all included studies (34 studies) [80-113]
